# Valorization of *Ficus carica* Pruning Residues as Selective Botanical Insecticides: Optimized Furanocoumarin Extraction, Efficacy Against Neotropical Stink Bugs, and Mechanistic Insights via Molecular Docking

**DOI:** 10.1002/arch.70200

**Published:** 2026-08-01

**Authors:** Thais A. Almeida, Arley R. Páez, Lara T. M. Costa, Guy Smagghe, Yara M. Cardoso, Letícia M. Faria, Eugênio E. Oliveira, João Paulo V. Leite

**Affiliations:** ^1^ Department of Biochemistry and Molecular Biology Federal University of Viçosa, University Campus Viçosa Minas Gerais Brazil; ^2^ Department of Entomology Federal University of Viçosa, University Campus Viçosa Minas Gerais Brazil; ^3^ Institute of Entomology Guizhou University Guiyang Guizhou China; ^4^ Department of Biology Vrije Universiteit Brussel (VUB) Brussels Belgium

**Keywords:** agricultural residues, bergapten, insecticidal activity, Moraceae, Pentatomidae, psoralen, target selectivity

## Abstract

The Neotropical stink bugs *Euschistus heros* and *Diceraeus melacanthus* are major pests of soybean and maize in South America, yet current chemical control strategies face widespread resistance, highlighting the urgent need for sustainable alternatives. This study investigates *Ficus carica* pruning residues as a source of selective botanical insecticides, integrating optimized extraction, phytochemical profiling, bioassays, and molecular modeling. Eight extracts were prepared under varying solvent, temperature, and acidity conditions, and analyzed for total phenolic content (TPC) and the key furanocoumarins psoralen and bergapten. Acidification enhanced overall mass yields, while mild ethanol extraction at room temperature selectively maximized furanocoumarin recovery, yielding psoralen concentrations up to 14.83 mg g^−1^, which is substantially higher than previously reported in leaves or fruit. Biological evaluation of the optimized ethanolic extract (ERA) revealed strong insecticidal activity, with 86% mortality of *E. heros* and 40% of *D. melacanthus* nymphs at 48 h, and a calculated LC_50_ of 1232 mg L^−1^ for *E. heros*. The differential susceptibility between species suggests both metabolic and cuticular factors influence efficacy. Computational docking and phylogenetic analyses suggested a potential mechanistic basis for the observed selectivity: furanocoumarins are predicted to bind hemipteran AChE via a compensatory polar scaffold, whereas binding to *Apis mellifera* AChE is predicted to be weaker due to lineage‐specific differences in aromatic density within the catalytic gorge, potentially explaining the minimal off‐target susceptibility. The molecular modeling results characterize these compounds as low‐affinity, reversible inhibitors, combining effective pest control with a favorable safety profile for pollinators. The present work demonstrates that valorizing agro‐industrial waste from *F. carica* can yield potent, selective, and environmentally safer insecticidal agents. The integration of extraction optimization, biological evaluation, and molecular modeling provides a robust framework for developing sustainable botanical insecticides, advancing circular economy principles in pest management and offering promising alternatives to synthetic neurotoxins.

## Summary

• Optimized extraction of *Ficus carica* branches yields up to 14.8 mg/g psoralen.
• The extract causes 86% mortality in *Euschistus heros* (LC_50_ = 1232 mg/L).
• Molecular docking confirms selective AChE inhibition in stink bugs, sparing honeybees.

## Graphical abstract

Optimized extracts from *Ficus carica* pruning waste exhibit strong biological activity against major stink bug pests. Complementary in silico analyses suggest this efficacy relies on pest‐specific acetylcholinesterase inhibition, predicting a promising, pollinator‐safe alternative for sustainable agriculture.

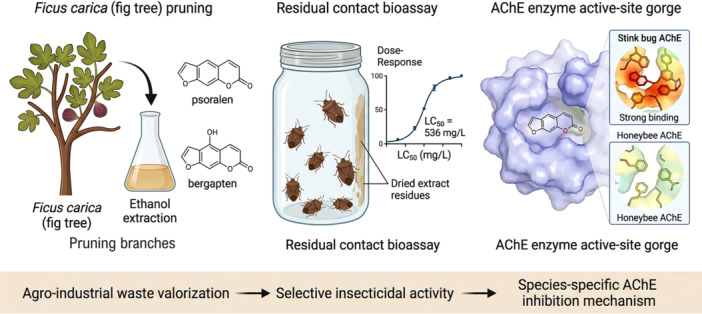

## 1 Introduction

The Neotropical brown stink bug *Euschistus heros* (Fabricius, 1798) and the green‐belly stink bug *Diceraeus melacanthus* (Fabricius, 1775) are among the most destructive insect pests affecting grain production in South America. In Brazil, the world's leading soybean producer, these highly polyphagous pentatomids cause severe economic losses in soybean (*Glycine max*) and maize (*Zea mays*), with annual damage estimated to exceed USD 10 billion (Moreira et al. 2024; Steinhaus et al. 2022). Their pest status is reinforced by intensive agricultural practices, such as no‐tillage systems and soybean–maize rotation, which create a continuous “green bridge” that facilitates year‐round population persistence. Current pest management relies heavily on chemical insecticides; however, *E. heros* populations have developed broad resistance to pyrethroids and neonicotinoids due to long‐term selection pressure (Tibola et al. 2021), while *D. melacanthus* shows increasing tolerance to these compounds (Somavilla et al. 2020). These trends highlight a critical need for alternative control strategies that are both effective and environmentally safer.

Botanical insecticides derived from plant secondary metabolites represent a promising avenue for sustainable pest management. Unlike conventional neurotoxic insecticides, complex botanical extracts may act on multiple molecular targets simultaneously, a feature that could reduce the likelihood of rapid resistance evolution. Among plant species with insecticidal potential, *Ficus carica* L. (Moraceae), commonly known as the fig tree, produces a diverse array of bioactive compounds, including phenolic acids, flavonoids, and furanocoumarins such as psoralen and bergapten, which have been implicated in plant defense against herbivory (Abdullah et al. 2025). Previous studies have demonstrated larvicidal and antifeedant activities of *F. carica* extracts against *Spodoptera frugiperda* and *Sitophilus oryzae*, with these effects being associated with interference in digestive enzymes and metabolic pathways (Kalpna et al. 2022; Alzahrani et al. 2024).

In a previous study from our laboratory, Britto et al. (2021) demonstrated the insecticidal potential of *F. carica* branch extracts against *E. heros* through bioassay‐guided fractionation of the alcoholic extract. Their phytochemical analysis indicated that the furanocoumarins psoralen and bergapten were the main compounds associated with the insecticidal activity of this extract, which exhibited an LC_50_ of 5.9 mg mL^−1^ against *E. heros* nymphs (Britto et al. 2021). Therefore, the present study builds on this previous work by shifting the focus from bioassay‐guided identification of active compounds to the optimization of extraction methods aimed at maximizing the recovery of psoralen and bergapten from *F. carica* branches.

Despite these promising attributes, important knowledge gaps remain. Existing studies have shown the biological activity of *F. carica* extracts against several insect pests, but they have not fully addressed how extraction conditions influence the chemical profile, furanocoumarin yield, and insecticidal efficacy of fig‐derived metabolites. Moreover, although psoralen and bergapten have been associated with activity against *E. heros*, their relevance in broader pentatomid management, including activity against *D. melacanthus*, remains insufficiently explored. Likewise, the molecular interactions underlying the selectivity and toxicity of these furanocoumarins in stink bugs require further investigation, limiting the rational development of *F. carica*‐derived compounds as agrochemical leads.

In this context, the present study addresses two central biological questions: (i) how do extraction parameters modulate the yield and composition of bioactive furanocoumarins from *F. carica* branches, and (ii) how do these compounds interact with molecular targets relevant to pentatomid physiology? By integrating optimized phytochemical extraction, bioassays against *E. heros* and *D. melacanthus*, and computational docking analyses, this work aims to provide preliminary computational insights into plant–insect chemical interactions and to support the development of *F. carica*‐based formulations as safer, plant‐derived alternatives for stink bug management.

## 2 Materials and Methods

### 2.1 Plant Material

Branches of *F. carica* (cv. Roxo de Valinhos) were collected in June 2023 from the experimental orchard of the Federal University of Viçosa (UFV), Minas Gerais, Brazil (20°45’14” S, 42°52’53” W). Sampling focused exclusively on pruning residues, thereby valorizing agricultural by‐products and aligning with circular economy and green chemistry principles. A voucher specimen was deposited at the VIC Herbarium (UFV) under registration number VIC 31703. Access to genetic heritage was authorized by the Brazilian National System for the Management of Genetic Heritage and Associated Traditional Knowledge (SisGen), under authorization number AF1E3D3.

After collection, the plant material was washed with distilled water, cut into small fragments, and dried in a forced‐air circulation oven at 40°C for 72 h. The dried biomass was then milled using a knife mill and passed through a 40‐mesh sieve to ensure homogeneous particle size. The resulting powder was stored in hermetically sealed containers, protected from light, at room temperature until extraction.

### 2.2 Preparation of Extracts

The preparation of extracts was adapted from the general methodology described by Britto et al. (2021), with modifications to implement a full factorial experimental design. A full factorial experimental design was applied to investigate the influence of solvent polarity, extraction temperature, and medium acidity on phytochemical recovery. Eight distinct extracts were prepared using 50 g of dried plant powder and 500 mL of solvent, corresponding to a solid‐to‐solvent ratio of 1:10 (w/v).

The extraction solvents consisted of absolute ethanol or methanol, either in neutral form or acidified with hydrochloric acid (HCl) to a final concentration of 0.05 M (pH: ~1.3). Two extraction conditions were evaluated: (i) Heat reflux extraction: Performed in round‐bottom flasks under continuous reflux at 60°C for 3 h. (ii) Room‐temperature extraction: Conducted in Erlenmeyer flasks under constant orbital agitation for 24 h at 25°C ± 2°C.

To ensure exhaustive extraction, each procedure was repeated once using the residual plant biomass and fresh solvent under identical conditions. The resulting supernatants were combined, filtered through Whatman No. 1 filter paper, and concentrated under reduced pressure using a rotary evaporator. The concentrated extracts were subsequently lyophilized to obtain dry crude extracts.

**Figure 1:**
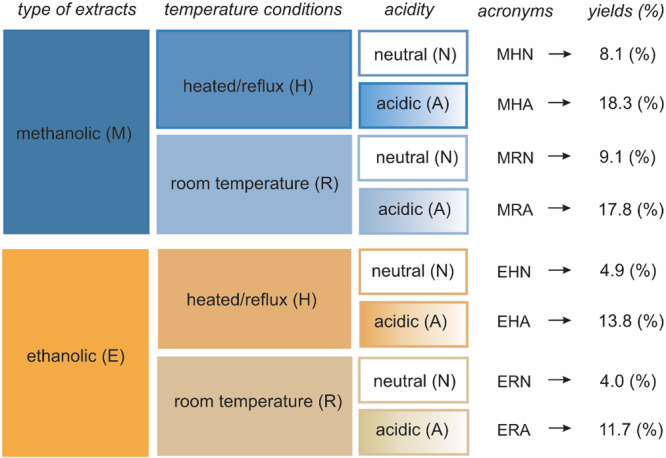
Yield (%, w/e) of *Ficus carica* branch extracts obtained by different extraction methods.

Extracts were coded using a three‐letter system indicating solvent type (E: ethanol; M: methanol), extraction temperature (H: heated/reflux; R: room temperature), and acidity (N: neutral; A: acidic). For example, ERA refers to an ethanolic extract obtained at room temperature under acidic conditions. Extraction yields and corresponding codes are summarized in (Figure 1). All dried extracts were stored in amber glass vials at −20°C until further phytochemical characterization and biological assays.

### 2.3 Determination of Total Phenolic Content (TPC)

The total phenolic content was quantified using the Folin–Ciocalteu colorimetric method adapted from Gontijo et al. (2017). Briefly, lyophilized extracts (~30 mg) were dissolved in approximately 15 mL of methanol in a 25 mL volumetric flask, and the volume was subsequently adjusted to exactly 25 mL with methanol to yield a final concentration of 1.2 mg mL^−1^. A 1.0 mL aliquot of the extract solution was transferred to a test tube containing 7.5 mL of distilled water and mixed with 0.5 mL of Folin–Ciocalteu reagent. After 3 min, 1.0 mL of sodium carbonate solution (7.5% w/v) was added to neutralize the reaction. The mixture was incubated in the dark at room temperature for 2 h, and absorbance was measured at 720 nm using a UV–VIS spectrophotometer (UV − 1800, Shimadzu, Kyoto, Japan). A calibration curve was constructed using gallic acid as the external standard (stock solution: 0.5 mg/mL; linear range: 0.5–7.5 μg mL^−1^ final concentration). Quantification was performed by linear regression analysis, and results were expressed as milligrams of gallic acid equivalents per gram of extract (mg GAE g^−1^). All measurements were performed in triplicate.

### 2.4 HPLC‐DAD Quantification of Psoralen and Bergapten

Chromatographic analyses were performed on a Shimadzu Prominence LC‐20AD system (Shimadzu, Kyoto, Japan), equipped with a CTO‐20A column oven and an SPD‐M20A diode array detector (DAD). The analytical method was based on the protocol of Britto et al. (2021), with minor modifications to improve peak resolution and separation efficiency. Analyses were carried out using a Shim‐pack VP‐ODS C18 column (5 µm, 4.6 × 150 mm), maintained at 40°C.

The mobile phase consisted of deionized water (solvent A) and HPLC‐grade acetonitrile (solvent B), delivered at a flow rate of 1.0 mL min^−1^. The gradient elution program was as follows: 0–8 min, 50% B; 8–9 min, 50%–95% B; 9–10 min, 95% B (isocratic); 10–11 min, 95%–50% B; followed by a 7 min re‐equilibration period, resulting in a total run time of 18 min. Detection was performed at 245 nm.

Samples were prepared by dissolving the extracts in methanol (5 mg mL^−1^), followed by filtration through 0.45 µm membrane filters prior to injection (10 µL). Analytical standards of psoralen and bergapten (≥ 99.0% purity, Sigma–Aldrich, St. Louis, MO, USA) were used for quantification. Calibration curves were constructed using six concentration levels for psoralen (5.5, 11.0, 22.0, 44.0, 88.0, and 104.5 µg mL^−1^) and eight levels for bergapten (0.59, 1.18, 2.36, 4.72, 9.44, 14.16, 18.88, and 28.32 µg mL^−1^). Linearity was confirmed by linear regression (*R* ^2^ > 0.99). Method sensitivity was evaluated through the Limit of Detection (LOD) and Limit of Quantification (LOQ), calculated as 3 and 10 times the signal‐to‐noise ratio, respectively. The resolution factor (Rs) between psoralen and bergapten peaks was calculated using the equation Rs = 2(tR^2^ − tR1)/(w1 + w2), where tR represents retention times and w represents peak base widths. Results were calculated using the external standard method and expressed as milligrams of compound per gram of dry extract (mg g^−1^).

### 2.5 Insecticide Bioassays

#### 2.5.1 Insect Rearing

Colonies of *E. heros* and *D. melacanthus* were obtained from a mass‐rearing facility maintained at the Laboratory of Invertebrate Physiology and Neurology, Federal University of Viçosa (UFV). Insects were reared under controlled conditions of 25°C ± 2°C, 60% ± 10% relative humidity (RH), and a photoperiod of 14 h light followed by 10 h darkness, following established rearing protocols (Santos et al. 2016; Castellanos et al. 2019; Rodrigues et al. 2021). The insects were housed in plastic containers and fed fresh green bean pods (*Phaseolus vulgaris* L.), peanuts (*Arachis hypogaea* L.), and sunflower seeds (*Helianthus annuus* L.).

Egg masses were collected from organza substrates placed in adult cages and transferred to Petri dishes containing green bean pods (~3 cm) until hatching. Nymphs were reared until the third instar, at which point they were transferred to larger containers. Colony maintenance was performed twice weekly.

#### 2.5.2 Toxicity Screening (Residual Contact Bioassay)

A discriminatory‐dose bioassay was conducted to evaluate the susceptibility of third‐instar nymphs of *E. heros* and *D. melacanthus* to the ERA extract using a residual contact (film) method. Briefly, 1 mL of extract solution (3000 mg L^−1^ in 96% ethanol) was applied to the inner surface of 250 mL glass jars, based on the protocol described by Moreira et al. (2024). Jars were rotated on a rotary shaker for 10 min to ensure uniform coating and complete solvent evaporation. Control jars received 1 mL of 96% ethanol only.

After drying, ten nymphs were introduced into each jar, which was then covered with organza fabric and secured with elastic bands. Five replicates were performed per species (*n* = 50 insects per treatment). Experimental units were maintained under the same environmental conditions as the rearing colonies. Mortality was assessed at 24 and 48 h post‐exposure, and insects were considered dead if they did not respond to gentle mechanical stimulation.

#### 2.5.3 Concentration‐Response Bioassay

Based on screening results, a concentration‐response bioassay was performed for *E. heros* to estimate lethal concentrations. The ERA extract was tested at five concentrations: 100, 300, 500, 1000, and 3000 mg L^−1^. The experimental procedures for jar coating, drying, and insect exposure followed the protocol described in Section 2.5.2. Ten replicates were conducted for each concentration and the control (*n* = 100 insects per concentration). Mortality was recorded at 24 and 48 h post‐treatment.

### 2.6 Molecular Mechanism Study

#### 2.6.1 Ligand Modelling and Preparation

Ligand modeling and structural preparation were performed following standard computational workflows (e.g., Tosco et al. 2011; Stewart 2013). Two‐ and three‐dimensional (3D) structures of the furanocoumarins psoralen, bergapten, and angelicin, as well as reference acetylcholinesterase (AChE) inhibitors (donepezil, galantamine, tacrine, huperzine A, and edrophonium), were generated using ACD/ChemSketch v2023.2.3 (Advanced Chemistry Development, Toronto, ON, Canada). Protonation states and predominant microspecies at physiological pH (7.4) were determined with MarvinSketch v25.1.0 (Chemaxon, Budapest, Hungary).

A stochastic conformational search was performed using Open3DALIGN v2.3 to identify global minimum‐energy conformers (Tosco et al. 2011). The protocol comprised 1000 independent molecular dynamics trajectories (1 ps at 1000 K, Δt = 1 fs), employing the MMFF94 force field with GBSA implicit solvation. The lowest‐energy conformers were converted to MOP format using Open Babel v3.1.1 (O'Boyle et al. 2011) and subjected to quantum‐mechanical refinement with MOPAC v22.1.0 using the PM7 semi‐empirical method (Stewart 1990; Stewart 2013). Geometry optimization was carried out using the Eigenvector‐Following (EF) algorithm, strict SCF convergence criteria, and COSMO implicit solvation (*ε* = 78.4). Closed‐shell systems were treated as singlets, while open‐shell species were optimized under the Unrestricted Hartree‐Fock (UHF) formalism. Ligand topology and atom typing were validated and standardized using the check_mol2 utility from the GOLD suite to ensure full compatibility with subsequent docking simulations.

#### 2.6.2 Structural Modeling and Preparation of AchE Proteins

The structural modeling and preparation of AChE proteins were performed using an automated computational pipeline based on established protocols (Edgar 2022; Abramson et al. 2024). As annotated AChE sequences for *E. heros* and *D. melacanthus* are not available, orthologous sequences from closely related Pentatomidae species were selected as surrogates. Candidate sequences were retrieved via NCBI Protein BLAST (Altschul et al. 1990), using the *Anopheles gambiae* AChE crystal structure (PDB ID: 5 × 61) as the query. Sequences from *Halyomorpha halys* (GenBank: XP_014279463.1) and *Nezara viridula* (GenBank: CAH1398876.1) were selected based on high homology criteria (Max Score > 500; Query Cover ≥ 97%; Identity > 70%). An *Apis mellifera* AChE sequence (GenBank: ANT80563.1) was additionally included to support selectivity analysis. Functional domain annotation was confirmed using InterPro (Paysan‐Lafosse et al. 2023).

3D protein structures were predicted using the AlphaFold3 server (Abramson et al. 2024). The resulting models were refined using the AMBER relaxation protocol implemented in the ColabFold framework (Mirdita et al. 2022). Energy minimization was performed with the OpenMM engine (Eastman et al. 2017) under GPU acceleration, using a convergence tolerance of 2.39 kcal mol^−1^, a maximum of 2500 iterations, and positional restraints of 10 kcal mol^−1^ Å^−1^. Structural quality was evaluated before and after refinement using the SWISS‐MODEL Structure Assessment tools (Waterhouse et al. 2018).

For comparative purposes, experimentally resolved AChE structures were retrieved from the PDB‐REDO databank (Joosten et al. 2014), including *Drosophila melanogaster* (PDB IDs: 6XYS, 6XYU, 6XYY), *Torpedo californica* (PDB IDs: 1DX6, 1EA5, 1GPK, 1W6R, 6EUE, 6H14, 1ACJ, 1AX9), and *Homo sapiens* (PDB IDs: 4EY4, 4EY5, 4EY7, 5HF5, 6O4W, 6WUZ). Structures were standardized using pdb‐tools v2.3.1 (Rodrigues et al. 2018) to remove solvent molecules and heteroatoms, resolve alternative conformations, and renumber residues.

Multiple sequence alignment was performed with MUSCLE v5.3 (Edgar 2022). Pairwise identity and similarity matrices (BLOSUM62) were calculated using a custom Python script based on Biopython (Cock et al. 2009) to ensure full reproducibility. Structural consistency was assessed by superposing all models onto the high‐resolution human AChE reference (PDB ID: 4EY7) using LovoAlign v20.0.2.27 (Martínez et al. 2007). Models with RMSD values exceeding 2.0 Å were excluded. Final receptor preparation was automated via a Bash pipeline integrating PDB2PQR v3.7.1 (Jurrus et al. 2018) and REDUCE v4.10 (Word et al. 1999), assigning protonation states at pH: 7.2 and optimizing hydrogen‐bond networks consistent with the AMBER force field.

#### 2.6.3 Molecular Docking Simulation

Molecular docking simulations were performed using protocols adapted for GOLD v2024.3.0 based virtual screening (Jones et al. 1997; Verdonk et al. 2003), employing a genetic algorithm‐based conformational sampling strategy with a dual‐scoring approach. The docking search space was defined based on the aligned structural ensemble. To locate the conserved active‐site region, coordinates of co‐crystallized inhibitors from the reference dataset, previously superposed using LovoAlign, were used to calculate a consensus centroid encompassing both the catalytic active site (CAS) and the peripheral anionic site (PAS), structurally defined according to Cheung et al. (2012).

The binding site was defined as a sphere with a 10 Å radius centered at *x* = −13.7, *y* = −44.5, and *z* = 28.8. Ligand conformational sampling employed the Genetic Algorithm (GA) with automatic efficiency settings (autoscale = 2). Initial pose generation and ranking used the GoldScore function (Jones et al. 1997), followed by rescoring with ChemScore (Eldridge et al. 1997). Implicit solvation was enabled (solvate_all = 1), and torsional sampling was guided by Cambridge Structural Database distributions (use_tordist = 1).

For each ligand‐receptor pair, the top ten poses ranked by consensus score were retained. Final complex geometries were exported in PDB format using the gold_utils utility. Protein‐ligand interactions were analyzed using Flare v8.0.0 (Cresset, Litlington, UK) to generate two‐dimensional (2D) interaction diagrams and three‐dimensional interaction maps, including hydrogen bonding, hydrophobic contacts, and π‐π interactions. Additional structural inspection and geometric analyses were conducted using UCSF Chimera v1.19 (Pettersen et al. 2004) and Mol* Viewer v2.5 (Sehnal et al. 2021).

### 2.7 Statistical Analysis

#### 2.7.1 Experimental Data Analysis

Quantitative data from phytochemical analyses (TPC, HPLC‐DAD) are reported as the mean ± standard error (SE) of three independent replicates. Differences among extraction methods were evaluated using one‐way analysis of variance (ANOVA) in R v4.5.0. When significant F‐values were obtained, means were separated using Tukey's Honest Significant Difference (HSD) post‐hoc test (*α* = 0.05).

For insecticidal bioassays, mortality rates were corrected using Abbott's formula when control mortality ranged between 5% and 20%. Concentration‐response relationships were modeled using Probit regression analysis implemented in SigmaPlot v14.0 (Systat Software, San Jose, CA, USA) to estimate lethal concentrations (LC_50_ and LC_90_) with 95% confidence intervals (CI). Statistical significance was defined at *α* = 0.05.

#### 2.7.2 Docking Validation and In Silico Analysis

The reliability of the molecular docking protocol was assessed through redocking validation. The geometric accuracy of GOLD‐predicted ligand poses relative to their crystallographic reference conformations was quantified by calculating the root‐mean‐square deviation (RMSD) using LigRMSD v1.034 (Velázquez‐Libera et al. 2020).

For retrospective quantitative evaluation, experimental inhibitory potencies were retrieved from the ChEMBL database (Mendez et al. 2019) and manually curated. Reported half‐maximal inhibitory concentrations (IC_50_) were converted to inhibition constants (K_i_) using the Cheng–Prusoff equation, (Yung‐Chi and Prusoff 1973) incorporating the original kinetic parameters ([S] and Km). K_i_ values were subsequently transformed into experimental binding free energies (ΔGexp) according to the thermodynamic relationship: ΔGexp = RTln(K_i_) where R is the universal gas constant (1.987 cal mol^−1^ K) and T is absolute temperature (298 K).

All statistical analyses were conducted using R v4.5.0 (R Core Team, 2025) within the RStudio environment v2025.09.2 (Posit Software, PBC). Correlations between experimental ΔG_exp and predicted docking scores were evaluated using Pearson's product‐moment coefficient (*r*) and linear regression. Variations in predicted binding affinities across biological contexts were analyzed using two‐way ANOVA, with “Species” and “Ligand” as independent factors, followed by Tukey's HSD test (*p* < 0.05). Assumptions of normality and homoscedasticity were verified using Shapiro–Wilk and Levene's tests, respectively. Data visualization was performed using the ggplot2 and ggrepel packages.

## 3 Results and Discussion

### 3.1 Extraction Yields and TPC

Extraction efficiency of *F. carica* branches was significantly influenced by solvent polarity, temperature, and medium acidity (Figure 1). The highest mass yield (18.3%) was achieved using the methanolic extract under heating with HCl addition (MHA), closely followed by the room‐temperature acidified methanolic extract (MRA: 17.8%). Overall, acidification was the most critical factor for enhancing biomass recovery. All acidified extracts exhibited markedly higher yields than their neutral counterparts. For example, the acidified ethanolic extract under heating (EHA) yielded 13.8%, whereas the neutral version (EHN) produced only 4.9%. The other extracts followed a similar trend: the methanol room‐temperature neutral (MRN) and methanol room‐temperature acidified (MRA) extracts yielded 9.1% and 17.8%, respectively; the ethanolic room‐temperature neutral (ERN) and ethanolic room‐temperature acidified (ERA) extracts yielded 4.0% and 11.7%, respectively; while the methanol heated neutral (MHN) extract yielded 8.1%. This pattern suggests that acid‐mediated hydrolysis facilitates cell wall disruption and promotes the breakdown of furanocoumarin glycosides (Dai and Mumper 2010). Methanol consistently outperformed ethanol, likely due to its higher polarity (Ayandiran Aina and Fagbemi 2022). Notably, the substantial yields obtained at room temperature with acid (e.g., MRA) indicate that chemical hydrolysis can effectively substitute thermal energy, aligning with green chemistry principles by reducing energy input and improving sustainability.

**Figure 2:**
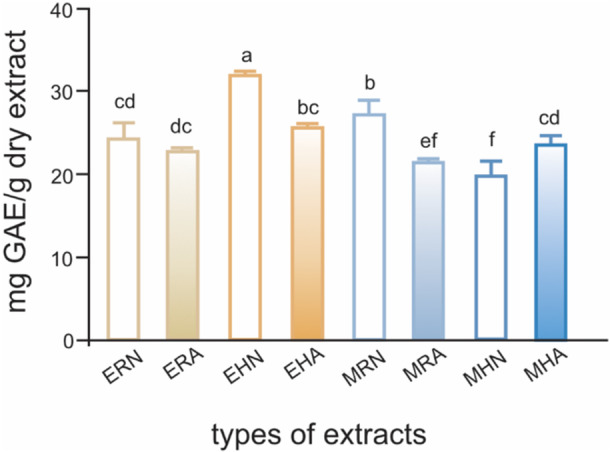
Total phenolic content in *Ficus carica* branch extracts. Values are expressed as mg of gallic acid equivalents (GAE) per gram of dry extract (mg GAE g^−1^) and represent the mean ± standard error. Different letters indicate statistically significant differences among mean values (*p* < 0.05) according to Tukey's test.

TPC displayed a different trend. The neutral ethanolic extract under heating (EHN) exhibited the highest concentration (32.50 ± 0.15 mg GAE g^−1^), significantly exceeding most other extracts (*p* < 0.05; Figure 2 and Table S1). Phenolic recovery grouped extracts into high (EHN), intermediate (MRN, EHA, ERN, and MHA; 25–28 mg GAE g^−1^), and low (ERA, MRA, MHN; 20–24 mg GAE g^−1^) categories. Interestingly, while acidification enhanced total mass yield, it did not necessarily maximize phenolic content, indicating that EHN conditions are more selective for phenolic compounds. These findings align with prior studies: Park et al. (2013) reported 47.30 mg GAE g^−1^ in ethanol extracts of *F. carica* branches, confirming that pruning residues are a valuable source of phenolics. Comparatively, fruits and leaves typically contain lower levels, ranging from 1.2 to 2.0 mg GAE g^−1^ in fruits (Ammar et al. 2015) and 10.7 to 31.0 mg GAE g^−1^ in leaves (Nadeem and Zeb 2018; Akhtar et al. 2019). This emphasizes the superior potential of branches. The selective recovery under EHN conditions demonstrates that careful optimization of solvent and temperature can valorize agro‐industrial waste into high‐value natural antioxidants.

In summary, the present study highlights the decoupling of extraction strategies for either maximizing total biomass yield or selectively enriching phenolics. The results demonstrate that room‐temperature acidified methanol efficiently recovers biomass, whereas neutral ethanol under mild heating selectively enriches phenolic compounds, offering tailored approaches for different industrial applications. However, we note that a detailed profiling of individual phenolic subclasses remains unexplored. Future studies could investigate structure–activity relationships of specific phenolics regarding antioxidant and insecticidal functions. Scaling these optimized extraction protocols for industrial applications while maintaining green chemistry principles warrants further exploration.

### 3.2 Quantification and Extraction Optimization of Psoralen and Bergapten

**Figure 3:**
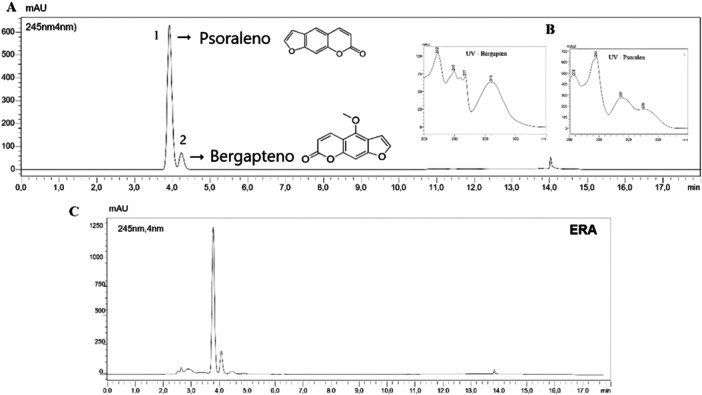
HPLC‐DAD analysis of psoralen and bergapten at 245 nm. (A) Chromatogram of the reference standards showing the separation of psoralen (peak 1) and bergapten (peak 2), alongside their chemical structures. (B) UV absorption spectra (200–400 nm) obtained for each compound, highlighting their characteristic absorption maxima used for confirmatory identification. (C) Representative chromatogram obtained for the ERA extract under the same chromatographic conditions.

HPLC‐DAD analysis successfully identified and quantified the furanocoumarins psoralen and bergapten in *F. carica* extracts. Chromatograms displayed two well‐defined peaks with retention times and UV spectra matching analytical standards (Figures 3 and S1). Method validation showed excellent linearity (*R* ^2^ > 0.998) and sensitivity, with a resolution factor of 1.63 between peaks, ensuring independent quantification without spectral overlap.

**Figure 4:**
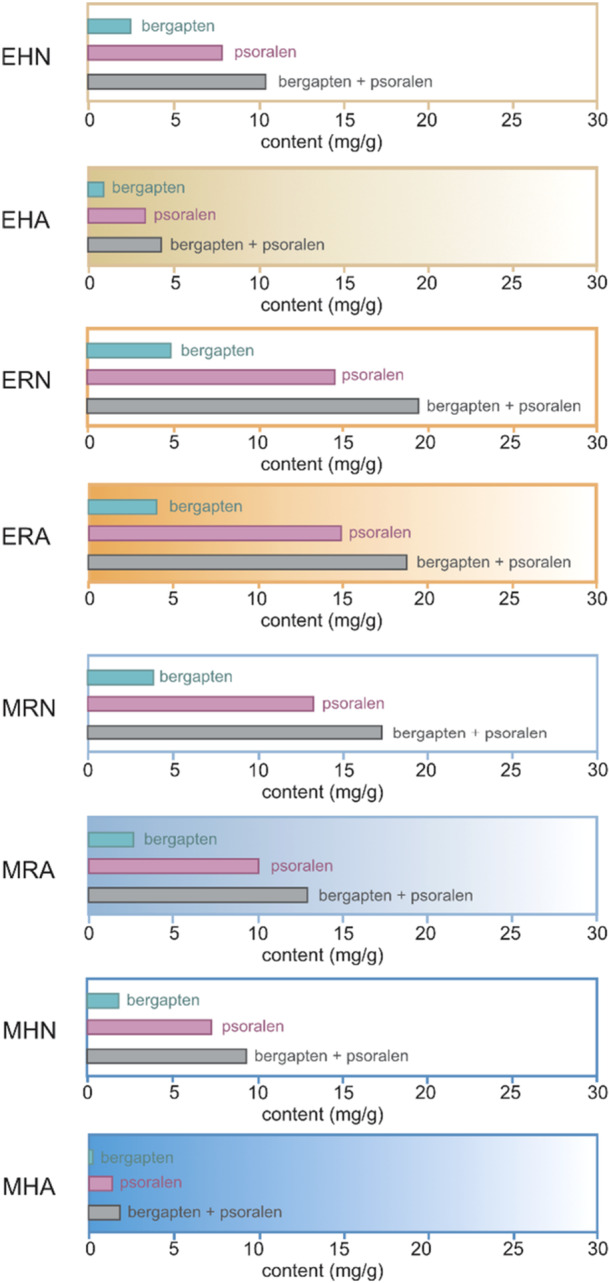
Levels of psoralen and bergapten (mg g^−1^) in branch extracts of *Ficus carica* were obtained under different extraction conditions.

Quantification revealed significant differences among extraction treatments (Figure 4). Extracts ERA and ERN yielded the highest psoralen concentrations (14.83 ± 0.15 mg g^−1^ and 14.60 ± 0.00 mg g^−1^, respectively). Regarding bergapten, ERN showed the highest content (4.95 ± 0.16 mg g^−1^), whereas heated methanolic extracts (MHA, EHA) produced the lowest levels. These concentrations markedly exceed previously reported values: Petruccelli et al. (2018) reported maximum levels of 1.49 mg g^−1^ psoralen and 0.62 mg g^−1^ bergapten in the “Portogallo” cultivar. Even high‐yielding varieties such as ‘Branca Tradicional’ (9.26 mg g^−1^ psoralen in leaves) (Oliveira et al. 2009) fall short of the branch‐based yields observed here. These results indicate that young branches are intrinsically richer sources of furanocoumarins than leaves or fruit peels and that specific extraction conditions applied here significantly enhance metabolite recovery.

Generalized Linear Model (GLM) analysis confirmed that furanocoumarin extraction was significantly influenced by solvent, temperature, and pH (*p* < 0.05), unlike total phenolics (Table S2). Temperature exerted the most pronounced effect, followed by pH and solvent. Superior yields obtained with room‐temperature ethanol highlight the thermolabile nature of these coumarin derivatives, (Elmusa and Elmusa 2024) suggesting that thermal stress may degrade the target compounds. Accordingly, mild extraction conditions, namely neutral pH, room temperature, and ethanol, are optimal for maximizing psoralen and bergapten recovery. This approach enhances sustainability by reducing energy consumption and solvent toxicity while preserving the pharmacological integrity of these bioactive molecules.

Together, this is the first systematic optimization of furanocoumarin extraction from *F. carica* branches, demonstrating that mild, neutral ethanol‐based extraction preserves thermolabile bioactives while achieving high yields. These findings have direct implications for the development of sustainable botanical insecticides and other bioactive formulations. However, it should be remarked that the stability of isolated furanocoumarins under storage and application conditions remains untested. The contribution of individual versus synergistic compounds to bioactivity requires further investigation. Additionally, scale‐up studies are needed to evaluate the feasibility of industrial extraction processes under green chemistry constraints.

### 3.3 Insecticidal Activity of ERA Extract Against *E. heros* and *D. melacanthus*

**Figure 5:**
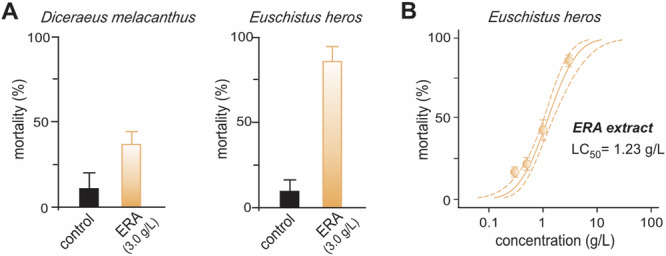
Susceptibilities of stink bug pests to the dried residues of the richest psoralen‐containing *Ficus carica* extract. The ERA denotes the ethanolic extract of *F. carica* obtained at room temperature under acidic conditions. (A) Mortality levels (%) of *Diceraeus melacanthus* and *Euschistus heros* when exposed to a discriminatory concentration (3 g L^−1^) of ERA extract. (A) Estimated lethal concentrations of ERA extract towards *E. heros*. Symbols represent observed mortality (mean ± SE) at each concentration. The solid line corresponds to the probit‐fitted mortality curve, and dashed lines denote the 95% confidence intervals.

The ethanolic extract ERA, selected for its high psoralen content, exhibited pronounced insecticidal activity via contact exposure. After 48 h, ERA caused 86% mortality in third‐instar nymphs of *E. heros* and 40% mortality in *D. melacanthus* (Figure 5). Probit analysis for *E. heros* revealed an LC_50_ of 1232 mg L^−1^ (95% CI: 1030–1543) and an LC_90_ of 4300 mg L^−1^ (95% CI: 3009–7645) (Figure 5), indicating a potency 4–10 times higher than previous reports for *F. carica* ethanolic extracts (LC_50_ 5900–14,100 mg L^−1^, Britto et al. 2021). This enhanced efficacy is likely driven by the elevated psoralen‐to‐bergapten ratio in ERA combined with direct contact exposure, which facilitates rapid uptake of active principles.

The observed bioactivity aligns with the established neurotoxic mechanism of linear furanocoumarins. Experimental evidence confirms that compounds like psoralen and bergapten directly inhibit AChE, interfering with cholinergic neurotransmission in various organisms (Britto et al. 2021; Guo et al. 2016; Xia et al. 2023). The steep dose‐response slope (LC_90_/LC_50_ ~ 3.5) suggests a relatively homogeneous population response, where physiological defense thresholds are quickly exceeded (Robertson et al. 2017). Although bergapten is often susceptible to P450‐mediated metabolism (Hung 1997), the pronounced toxicity suggests potential synergistic effects of the complex extract matrix. Co‐occurring matrix compounds, including flavonoids, may act as synergists by inhibiting detoxification enzymes such as P450s and GSTs, saturating metabolic capacity and prolonging the lethal persistence of furanocoumarins (Berenbaum and Zangerl 1993; Wen et al. 2006). This highlights the theoretical advantage of using complex botanical extracts to delay the development of resistance compared to single‐compound formulations. However, it is important to explicitly note that the biological assays in this study were conducted using the crude optimized extract (ERA) rather than isolated pure furanocoumarins. While the extract is highly enriched in psoralen and bergapten, we cannot discard the contribution of other minor secondary metabolites or these synergistic matrix effects in the overall observed mortality. Future bioassays evaluating isolated psoralen and bergapten, both individually and in combination, are necessary to determine their distinct individual toxicities and definitively establish the precise nature of these synergistic interactions.

Differential susceptibility between species (*E. heros* > *D. melacanthus*) likely reflects biochemical and morphological divergences. *Euschistus heros* exhibits variable resistance linked to metabolic gene expression (Lira et al. 2023), whereas *D. melacanthus* displays higher baseline tolerance (Somavilla et al. 2020). Although ultrastructural data for pentatomids are scarce, it is plausible that cuticular protein composition contributes to reduced penetration of lipophilic xenobiotics. In other insects, overexpression of structural proteins reinforces the chitin–protein matrix, impeding cuticular absorption (Huang et al. 2018; Li et al. 2025). Such a barrier may partially explain the lower mortality observed in *D. melacanthus*.

In summary, the present study demonstrates that branch‐derived ERA extract provides significantly enhanced insecticidal potency compared to prior *F. carica* extracts, emphasizing the importance of optimizing furanocoumarin content and leveraging synergistic matrix effects. The findings suggest that complex botanical formulations can overcome metabolic detoxification mechanisms and improve efficacy against pest populations. But the mechanistic basis of differential susceptibility between species remains non‐characterized. Therefore, we suggest that future studies should explore cuticular penetration, metabolic detoxification, and potential synergistic interactions in more detail. Additionally, field‐level efficacy, residual activity, and non‐target safety assessments are needed to translate these findings into practical, safer pest control strategies. Understanding the stability and bioavailability of furanocoumarins in agricultural environments will further inform sustainable application protocols. From a practical standpoint, the insecticidal efficacy of the ERA extract demonstrated under laboratory conditions warrants further research on its persistence in agricultural environments, its comparative performance against commercial insecticide standards, formulation feasibility (such as encapsulation to protect bioactive compounds from UV degradation), and selective field application strategies to minimize potential exposure of beneficial non‐target organisms.

### 3.4 Computational Docking and Active‐Site Sequence Comparison

Molecular docking simulations predicted that psoralen and bergapten exhibit moderate binding affinities across the analyzed AChEs, with predicted free energies ranging from −5.95 to −7.23 kcal mol^−1^ (Table 1).

**Table 1:** Binding affinity energies of furanocoumarins and reference inhibitors against the AChEs of six different species.

Ligand	*Apis mellifera*	*Drosophila melanogaster*	*Halyomorpha halys*	*Homo sapiens*	*Nezara viridula*	*Torpedo californica*
l‐Huperzine‐A	−8.62 ± 0.16^(c)A^	−8.21 ± 0.89^(bc)A^	−8.47 ± 0.14^(cd)A^	−8.41 ± 0.52^(c)A^	−8.53 ± 0.08^(d)A^	−7.98 ± 0.38^(d)A^
(‐)‐Galantamine	−8.18 ± 0.78^(bc)AB^	−9.60 ± 1.40^(c)B^	−8.29 ± 0.29^(c)AB^	−8.32 ± 0.40^(c)AB^	−7.82 ± 0.71^(cd)A^	−7.97 ± 0.49^(d)A^
2R‐Donepezil	−8.95 ± 0.12^(cd)A^	−12.11 ± 0.62^(d)D^	−9.10 ± 0.78^(d)A^	−11.06 ± 0.47^(d)CD^	−9.42 ± 0.71^(e)AB^	−10.36 ± 0.65^(e)BC^
2S‐Donepezil	−9.58 ± 0.37^(d)A^	−12.05 ± 0.88^(d)C^	−10.10 ± 0.51^(e)AB^	−10.98 ± 0.68^(d)BC^	−9.95 ± 0.49^(e)AB^	−10.53 ± 0.60^(e)AB^
Angelicin	−6.38 ± 0.45^(a)A^	−7.24 ± 0.06^(ab)A^	−6.51 ± 0.08^(b)A^	−6.81 ± 0.50^(b)A^	−6.56 ± 0.16^(b)A^	−6.89 ± 0.71^(bc)A^
Bergapten	−6.22 ± 0.20^(a)A^	−6.87 ± 0.27^(ab)AB^	−6.61 ± 0.36^(b)AB^	−6.91 ± 0.36^(b)B^	−6.75 ± 0.17^(b)AB^	−6.27 ± 0.34^(ab)A^
Edrophonium	−5.78 ± 0.32^(a)A^	−5.90 ± 0.57^(a)A^	−5.46 ± 0.16^(a)A^	−5.62 ± 0.17^(a)A^	−5.59 ± 0.25^(a)A^	−5.58 ± 0.25^(a)A^
Psoralen	−5.95 ± 0.44^(a)A^	−7.23 ± 0.36^(ab)B^	−6.52 ± 0.13^(b)AB^	−6.96 ± 0.45^(b)B^	−6.53 ± 0.08^(b)AB^	−6.62 ± 0.69^(bc)AB^
Tacrine	−7.42 ± 0.19^(b)A^	−8.33 ± 0.58^(bc)A^	−7.10 ± 0.12^(b)A^	−7.67 ± 0.64^(bc)A^	−7.21 ± 0.09^(bc)A^	−7.61 ± 1.13^(cd)A^

*Note:* Values represent the mean ± standard deviation (SD) of energies (kcal mol^−1^) obtained using GOLD software v2024.3.0. Lowercase letters in parentheses indicate statistically significant differences among ligands within the same organism (column comparison). Uppercase letters indicate statistically significant differences among species for the same ligand (row comparison).

**Figure 6:**
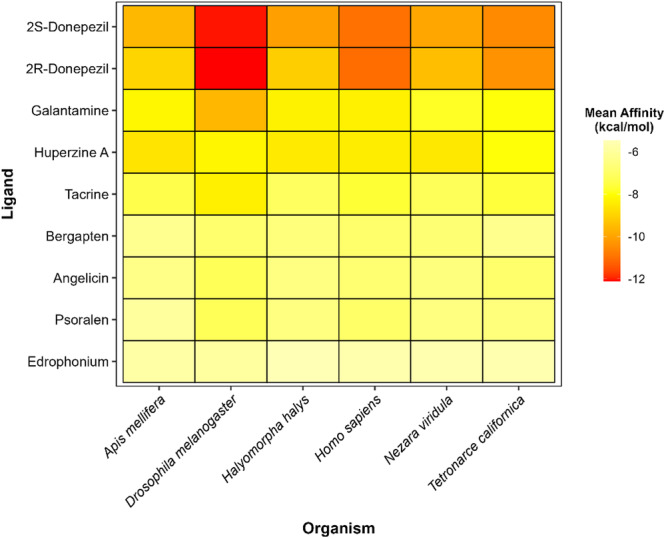
Mean binding affinity heatmap of furanocoumarins and reference inhibitors against AChE across six species. The color gradient represents the binding energy (kcal mol^−1^) calculated using GOLD v2024.3.0. The yellow spectrum corresponds to lower affinity values, while the orange‐to‐red spectrum indicates high‐affinity interactions.

These computational results are consistent with prior experimental data: psoralen inhibits crude brain AChE from *Rattus norvegicus* (IC_50_ ≈ 2 mM; ≈−4.7 kcal mol^−1^, Somani et al. 2015) and displays higher potency against *Electrophorus electricus* (IC_50_ = 6.48 μM; ≈−7.3 kcal mol^−1^, Ben Salem et al. 2013) Comparative analysis across species revealed a clear toxicity hierarchy, summarized in the mean‐affinity heatmap (Figure 6).

*Apis mellifera* was predicted to exhibit the weakest interactions (Group A; −5.95 to −6.22 kcal mol^−1^), suggesting a potentially lower off‐target risk for this pollinator. In contrast, agricultural pests (*N. viridula* and *H. halys*) showed intermediate affinities (Group AB), stronger than the honeybee but lower than *D. melanogaster* and *H. sapiens* (Group B). Benchmarking against commercial inhibitors revealed that furanocoumarins resemble reversible agents such as edrophonium in energetic profile but are weaker than high‐potency compounds like donepezil and huperzine A (> −8.0 kcal mol^−1^). This characterizes them as predicted low‐affinity, reversible inhibitors that are likely to act through sublethal modulation rather than acute cholinergic shock, although in vitro enzyme inhibition assays are required to validate these binding profiles.

Sequence comparisons provide structural rationale for these affinity differences. The catalytic triad (S200, E199, H438) and Trp83(W) are highly conserved (Table 2), preserving basal enzymatic function. However, key variations in the PAS and gorge entry modulate ligand recognition. Hemipterans and hymenopterans exhibit a lower aromatic density in their catalytic gorge compared to the vertebrate models. For instance, vertebrate residues capable of π‐stacking (Phe286, Tyr69) are replaced by smaller, non‐aromatic residues (Cys286, Ile69) in insects (Table 2). Phylogenetic analysis (Figure S2) and pairwise matrices (Tables S3 and S4) confirm that hemipteran pests and *A. mellifera* form a cohesive evolutionary cluster (67% identity, ~89%–90% similarity), distinct from vertebrate models (< 47% identity). These substitutions explain the lower binding energies observed in agricultural pests, whereas *D. melanogaster* maintains a peripheral “aromatic trap” (Met121, Tyr71), accounting for its vertebrate‐like affinity despite evolutionary distance.

**Table 2:** Multiple sequence alignment of the AChE active site residues lining the catalytic gorge.

Organism	Amino acid active site																	
	Y69	D71	W83	G117	G118	Y121	E199	S200	W277	S284	V285	F286	F288	Y328	F329	Y332	H438	G439
*Apis mellifera*	I	D	W	G	G	Y	E	S	W	G	I	C	F	Y	F	Y	H	A
*Drosophila melanogaster*	E	Y	W	G	G	M	E	S	W	G	I	L	F	Y	F	Y	H	G
*Halyomorpha halys*	I	D	W	G	G	Y	E	S	W	G	I	C	F	Y	F	Y	H	G
*Homo sapiens*	Y	D	W	G	G	Y	E	S	W	S	V	F	F	Y	F	Y	H	G
*Nezara viridula*	I	D	W	G	G	Y	E	S	W	G	I	C	F	Y	F	Y	H	G
*Torpedo californica*	Y	D	W	G	G	Y	E	S	W	S	I	F	F	F	F	Y	H	G

*Note:* The amino acid positions are numbered according to the *Homo sapiens* reference structure (PDB ID: 4EY7).

**Figure 7:**
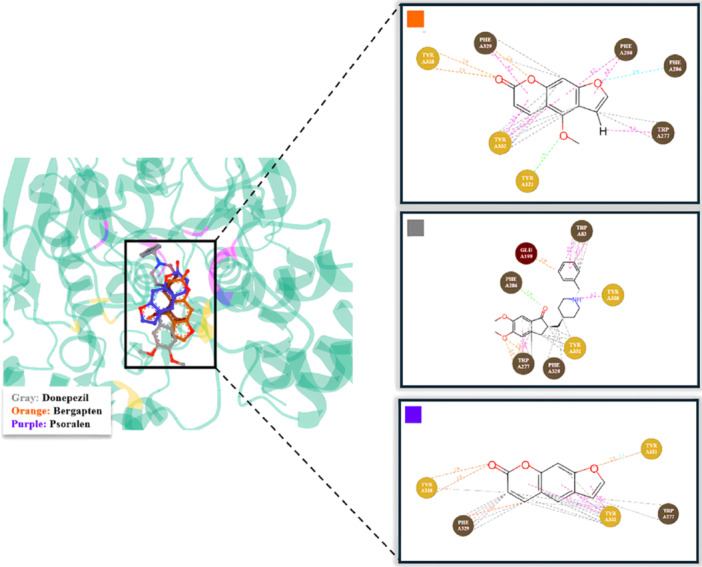
Structural analysis of Donepezil, Bergapten, and Psoralen binding in AChE (PDB: 4EY7). (Left) 3D view of the superimposed ligands docked within the AChE active site. Key protein regions are highlighted: yellow indicates the PAS and magenta indicates the CAS. (Right) 2D diagrams detailing ligand‐residue interactions. Interaction color code dashed lines: green/blue = strong/weak hydrogen bonds; gray = hydrophobic contacts; magenta/purple lines = π‐π and cation‐π interactions; orange = steric clashes.

**Figure 8:**
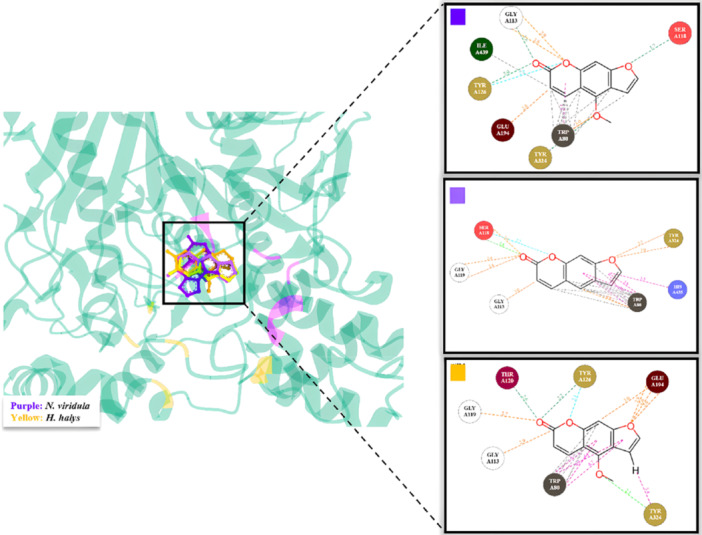
Structural analysis of bergapten and psoralen binding in AChE models of *Nezara viridula* (Purple) and *Halyomorpha halys* (Yellow). (Left) 3D view of the superimposed ligands docked within the AChE active site. Key protein regions are highlighted: yellow indicates the PAS and magenta indicates the CAS. (Right) 2D diagrams detailing ligand‐residue interactions. Interaction color code dashed lines: green/blue = strong/weak hydrogen bonds; gray = hydrophobic contacts; magenta/purple lines = π‐π and cation‐π interactions; orange = steric clashes.

Species‐specific interaction maps illustrate distinct binding modes driven by gorge topology. In the human model (Figure 7), furanocoumarins adopt a planar orientation stabilized by dense hydrophobic and π‐stacking interactions with Phe329, Tyr332, and Trp277. Donepezil, by contrast, anchors both the catalytic site and PAS via extensive cation‐π interactions, explaining superior affinity. In hemipteran pests, the absence of a vertebrate‐style aromatic cage necessitates a compensatory polar scaffold: ligands engage Tyr324 and Glu194, with species‐specific contributions such as Ser118 in *N. viridula* (Figure 8). This scaffold is less thermodynamically efficient, rationalizing moderate affinity energies.

**Figure 9:**
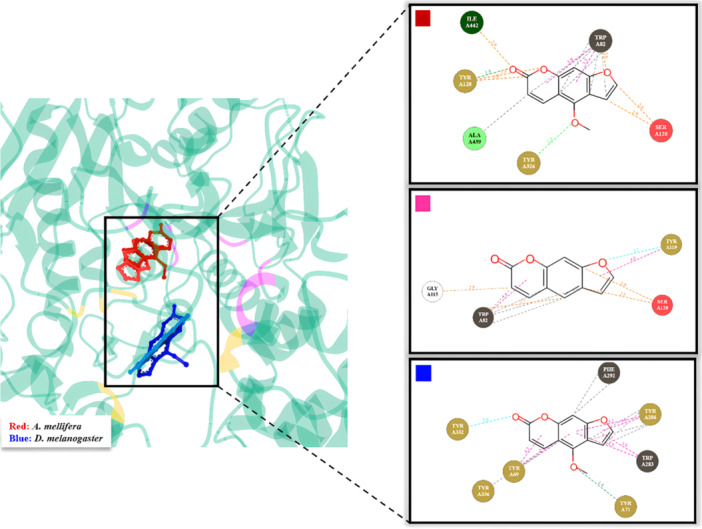
Structural analysis of Bergapten and Psoralen binding in AChE models of *Apis mellifera* (Red) and *Drosophila melanogaster* (Blue). (Left) 3D view of the superimposed ligands docked within the AChE active site. Key protein regions are highlighted: yellow indicates the PAS and magenta indicates the CAS. (Right) 2D diagrams detailing ligand‐residue interactions. Interaction color code dashed lines: green/blue = strong/weak hydrogen bonds; gray = hydrophobic contacts; magenta/purple lines = π‐π and cation‐π interactions; orange = steric clashes.

*Apis mellifera* shows a reduced number of functional aromatic residues in the binding pocket (Figure 9), relying primarily on Trp82 and short‐range hydrogen bonds (e.g., Tyr128 at 1.8 Å for bergapten) to stabilize ligands. This predicted reduction in hydrophobic and Van der Waals interactions is modeled to render the complex energetically labile, which aligns with the low mortality (< 12%) observed in previous studies. In contrast, *D. melanogaster* favors PAS binding at the gorge entrance, leveraging aromatic residues to compensate for limited deep‐site interactions. These observations reveal that susceptibility to furanocoumarins is dictated by species‐specific gorge architecture rather than phylogenetic proximity alone, providing a mechanistic explanation for selective toxicity.

Comparisons with Britto et al. (2021) on *E. heros* confirm convergent biological trends: reduced aromatic density in *A. mellifera* correlates with low ligand affinity, while pentatomids exploit polar scaffolds to compensate for weaker hydrophobic interactions. Despite methodological differences (AutoDock Vina vs. GOLD), interaction maps are consistent: conserved aromatic pillars and catalytic residues mediate ligand stabilization across models. The present study extends these insights by explicitly linking polar compensatory networks to selective pest inhibition.

The model stereochemistry was validated via MolProbity, yielding scores < 0.9, indicating sterically plausible structures. Ramachandran analysis confirmed > 96% of residues in favored regions (Figure S3). Re‐docking assays achieved RMSD < 1.0 Å (Table S5), and predicted docking scores correlated strongly with experimental affinities (Pearson's *r* = 0.94, *p* < 0.0001, *R* ^2^ = 0.89, Figure S4), confirming predictive reliability.

Taken together, the present study provides the first integrative mechanistic framework connecting furanocoumarin chemical structure, AChE active site architecture, and species‐selective toxicity. The elucidation of polar scaffold compensation in pests and aromatic loss in pollinators offers a rational basis for designing safer botanical insecticides. Despite robust in silico predictions, experimental validation of enzyme‐ligand kinetics in target pests and non‐target organisms is necessary. The influence of synergistic matrix compounds on AChE binding, metabolic degradation, and systemic uptake remains unexplored. Future research should integrate molecular dynamics simulations, in vivo neurophysiological assays, and field‐level evaluations to refine dosage and assess ecological safety. Additionally, exploring other furanocoumarin derivatives could identify candidates with optimized selectivity and potency, advancing sustainable pest management strategies.

We must explicitly emphasize that a primary limitation of this study is the absence of direct in vitro or in vivo biochemical assays (such as enzyme activity and kinetics measurements on target insect tissues) to confirm AChE inhibition. Furthermore, due to the lack of annotated genomic sequence data for *E. heros* and *D. melacanthus*, structural modeling relied on orthologous AChE sequences from related pentatomids (*H. halys* and *N. viridula*) as surrogates. While these surrogates share high homology, sequence variations in the binding cavity could affect docking scores and binding modes.

However, the potential of these compounds to act upon this target is strongly supported by existing empirical evidence. Previous biochemical evaluations have proven that both psoralen and bergapten inhibit AChE activity, as demonstrated in vitro against the pine wood nematode (*Bursaphelenchus xylophilus*) with recorded IC_50_ values of 564.59 μg mL^−1^ and 493.11 μg mL^−1^, respectively (Guo et al. 2016). More recently, the insecticidal efficacy of coumarins via AChE inhibition has been experimentally validated in agricultural pests like Spodoptera litura (Xia et al. 2023). Furthermore, previous toxicological and computational assessments have linked the insecticidal efficacy of furanocoumarin‐rich extracts to this specific mode of action (Britto et al. 2021). Therefore, while the binding modes and affinity hierarchies proposed here for *E. heros* represent computational hypotheses, they are grounded in established and experimentally validated biochemical interactions that warrant targeted experimental verification to establish the exact physiological target and confirm pollinator safety.

## 4 Conclusions

In conclusion, the optimization of extraction parameters from *F. carica* pruning residues demonstrated that room‐temperature ethanol extraction maximizes furanocoumarin recovery (psoralen and bergapten), yielding an extract (ERA) with insecticidal potential against the stink bug pests *E. heros* and *D. melacanthus* under laboratory conditions.

Integrating computational modeling suggested potential binding modes that may explain the observed species‐selective toxicity. Molecular docking and phylogenetic analyses predicted that furanocoumarins interact with hemipteran AChE through a compensatory polar scaffold, whereas binding affinity is predicted to be reduced in the pollinator *A. mellifera* due to a lower aromatic density in its catalytic gorge. These modeling results characterize *F. carica* furanocoumarins as potentially reversible, low‐affinity inhibitors, offering a useful hypothesis for their selective action.

The findings highlight the potential of *F. carica* residues as a source of botanical extracts for pest control. Future studies must focus on experimental validation of enzyme inhibition kinetics, environmental persistence, and field‐scale efficacy to support the development of stable formulations.

## Author Contributions

**Thais A. Almeida:** conceptualization, methodology, validation, investigation. **Arley R. Páez:** conceptualization, investigation, methodology, validation, software, formal analysis, data curation, supervision, visualization, writing – review and editing, writing – original draft. **Lara T. M. Costa:** investigation, methodology. **Guy Smagghe:** supervision, writing – review and editing, validation. **Yara M. Cardoso:** investigation, methodology. **Letícia M. Faria:** investigation, methodology. **Eugênio E. Oliveira:** supervision, writing – review and editing, funding acquisition. **João Paulo V. Leite:** supervision, writing – review and editing, validation, project administration, resources, funding acquisition.

## Ethics Statement

Ethical review and approval were waived for this study due to the nature of the biological models employed. The experiments involved invertebrate species (*Euschistus heros* and *Diceraeus melacanthus*), which are exempt from mandatory submission to the Ethics Committee on the Use of Animals (CEUA) according to Brazilian Law No. 11.794/2008.

## Conflicts of Interest

The authors declare no conflicts of interest.

## Supporting information


Supporting File

## Data Availability

Data sharing not applicable to this article as no datasets were generated or analysed during the current study.
