# Supplementary material for: Valorization of Ficus carica Pruning Residues as Selective Botanical Insecticides: Optimized Furanocoumarin Extraction, Efficacy Against Neotropical Stink Bugs, and Mechanistic Insights via Molecular Docking
Source: Arch Insect Biochem Physiol. 2026 Aug 1;122(4):e70200. doi: 10.1002/arch.70200 (PMC13428401; doi:10.1002/arch.70200)
Supplement: Supplementary file 1 — Supporting File [file ARCH-122-e70200-s001.docx]

**Valorization of *Ficus carica* Pruning Residues as Selective Botanical Insecticides: Optimized Furanocoumarin Extraction, Efficacy Against Neotropical Stink Bugs, and Mechanistic Insights via Molecular Docking**

Thais A. Almeida^1^ | Arley R. Páez^1^ | Lara T. M. Costa^2,3^ | Guy Smagghe^3,4^ | Yara M. Cardoso^1^ | Letícia M. Faria^1^ | Eugênio E. Oliveira^2^ | João Paulo V. Leite^1^

^1^Department of Biochemistry and Molecular Biology, Federal University of Viçosa, Avenida P.H. Rolfs, s/n, University Campus, Viçosa 36570-900, MG, Brazil | ^2^Department of Entomology, Federal University of Viçosa, Avenida P.H. Rolfs, s/n, University Campus, Viçosa 36570-900, MG, Brazil | ^3^Institute of Entomology, Guizhou University, Guiyang 550025, Guizhou, China | ^4^Department of Biology, Vrije Universiteit Brussel (VUB), 1050 Brussels, Belgium

**Correspondence:** Arley R. Páez (arley.paez@ufv.br); João Paulo V. Leite (jpvleite@ufv.br)

*Thais A. Almeida and Arley R. Páez contributed equally to this work.*

**Figure S1**. Representative HPLC-DAD chromatograms of *Ficus carica* branch extracts. The chromatograms demonstrate the retention times of the peaks related to psoralen and bergapten. The specific retention times identified for each extract were: ERA (3.990 and 4.285 min), MRA (3.962 and 4.255 min), EHA (3.937 and 4.216 min), MHA (3.917 and 4.191 min), ERN (3.914 and 4.186 min), MRN (3.904 and 4.171 min), EHN (3.907 and 4.171 min), and MHN (3.903 and 4.169 min).


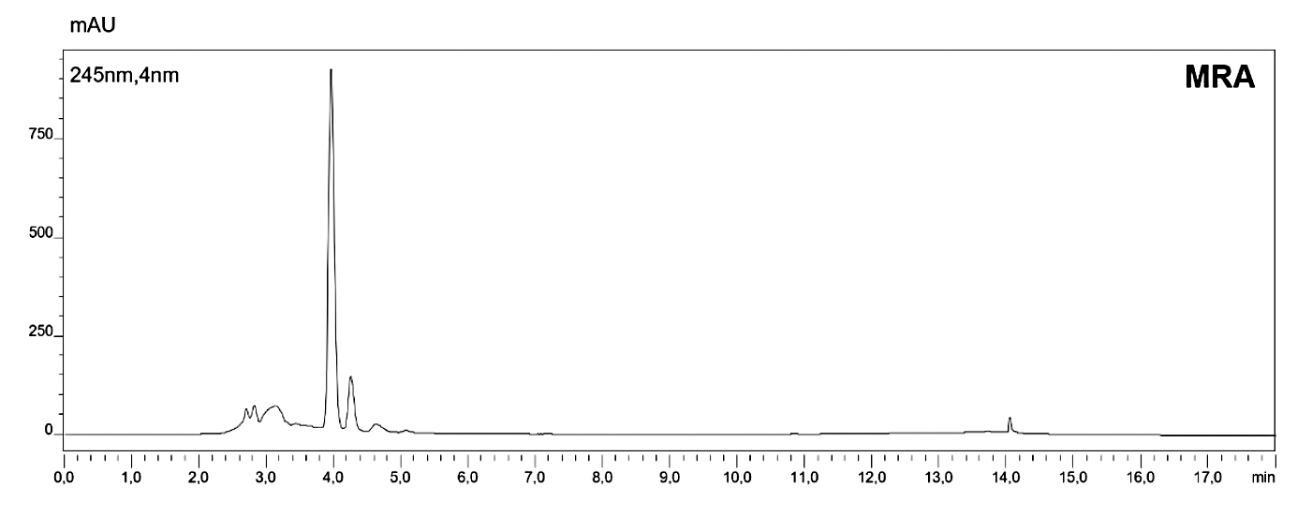

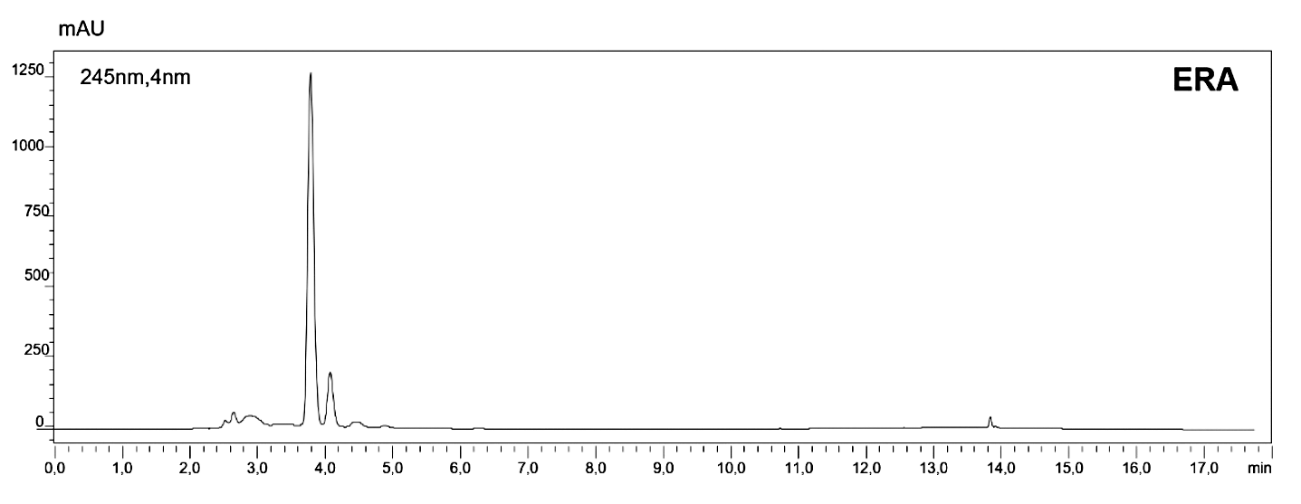


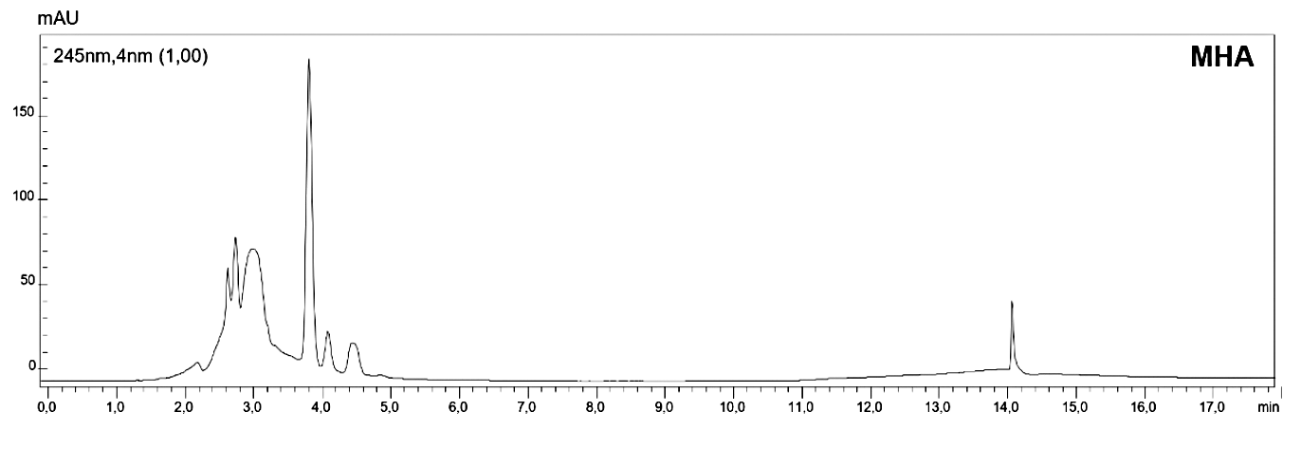


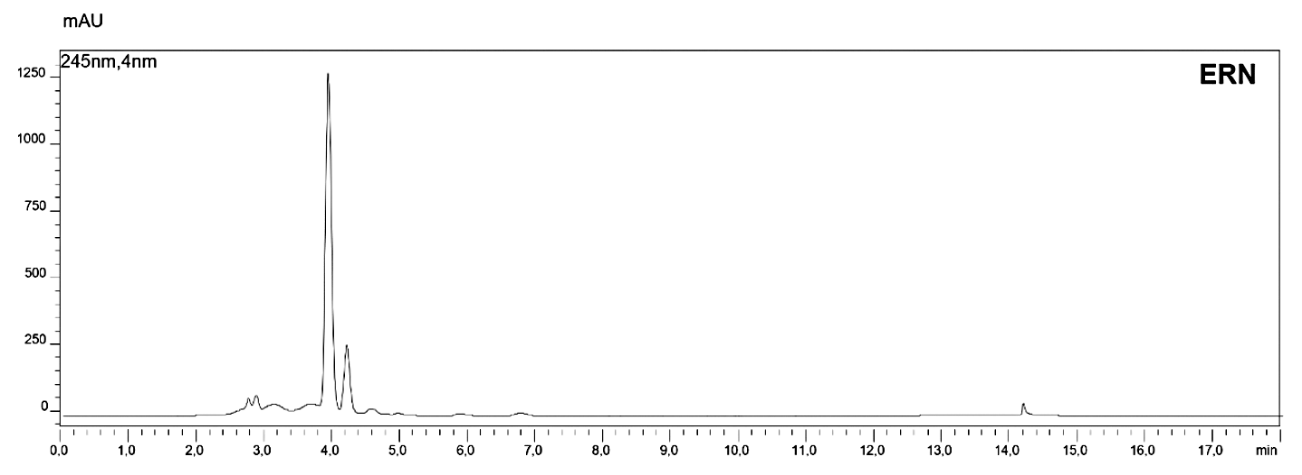


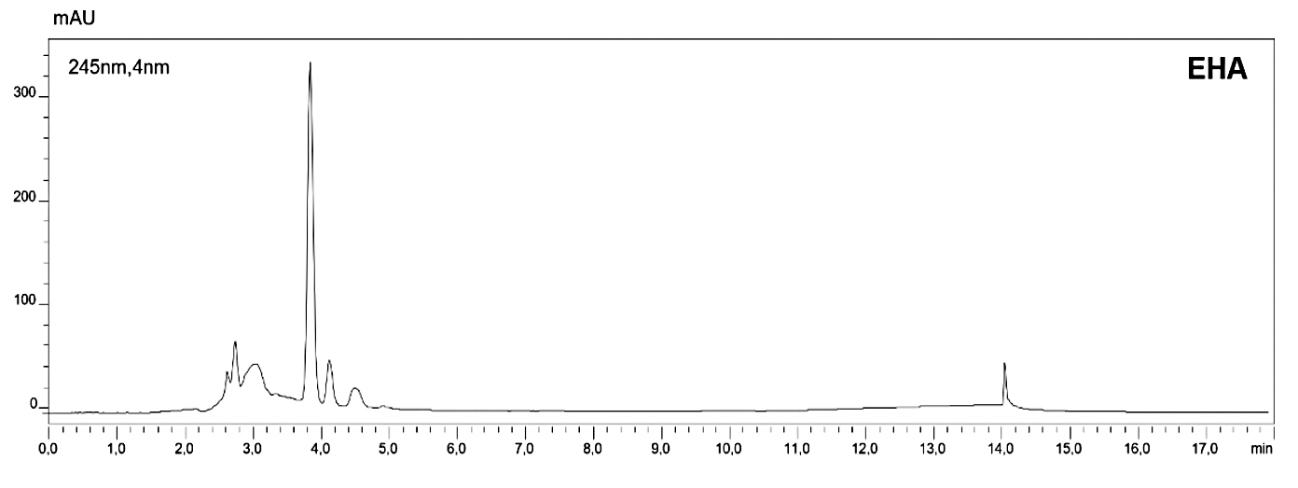


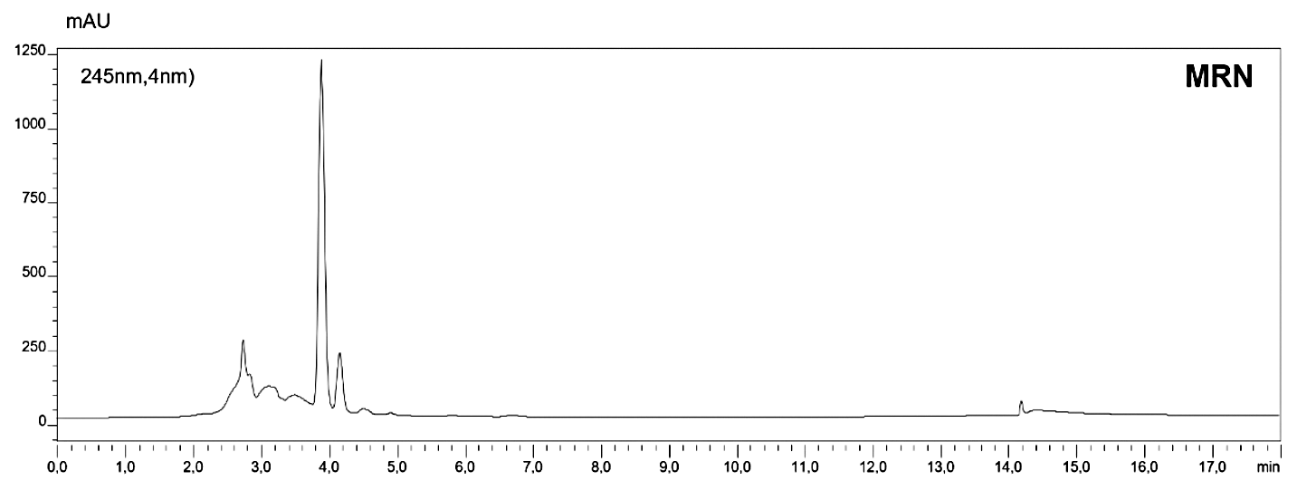


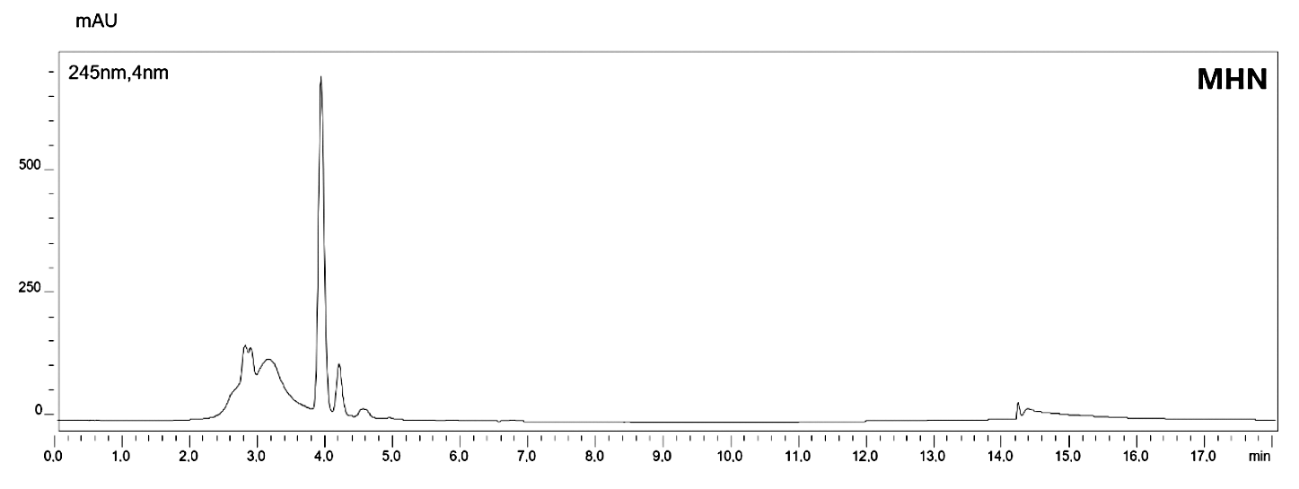


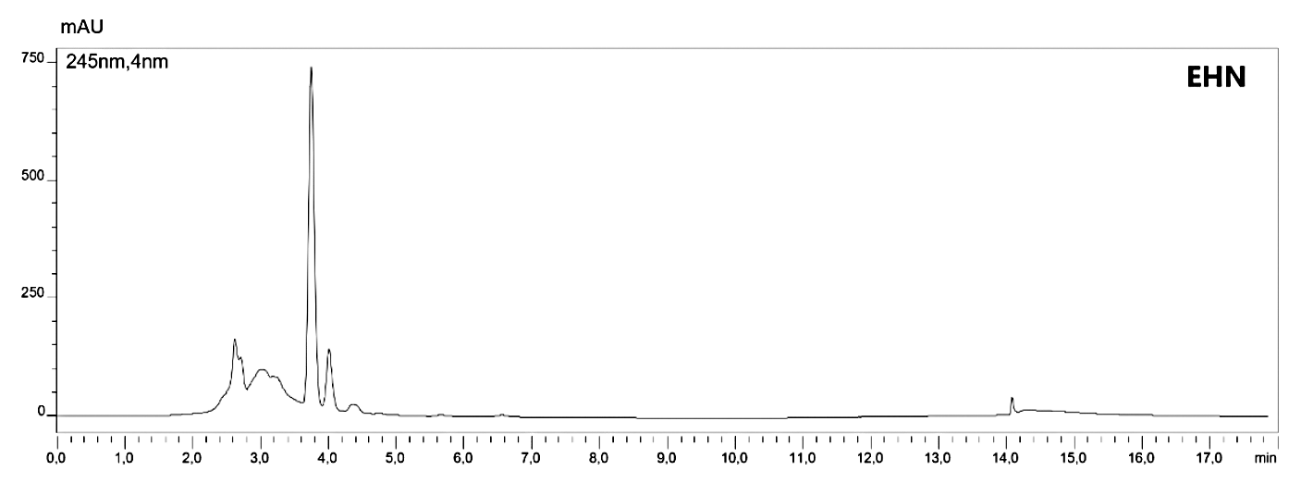


| **Extract Code** | **Total Phenolic Content (mg GAE g^-1^)** |
| --- | --- |
| EHN | 32.50 ± 0.15 |
| MHA | 27.40 ± 0.12 |
| EHA | 26.85 ± 0.24 |
| MRN | 25.20 ± 0.18 |
| ERN | 25.90 ± 0.31 |
| ERA | 22.10 ± 0.14 |
| MRA | 21.40 ± 0.25 |
| MHN | 20.80 ± 0.22 |

**Table S1.** Total Phenolic Content (TPC) of Ficus carica branch extracts obtained under different solvent, temperature, and pH conditions.

**Table S2.** Analysis of variance (GLM, SAS) for the effects of solvent, temperature, and pH on total phenolics, psoralen, and bergapten content.

| Response Variable | Source of Variation | df | | Sum of Squares (SS) | Mean Square (MS) | F-Value | p-value (Pr > F) | Significant |
| --- | --- | --- | --- | --- | --- | --- | --- | --- |
| Total Phenolics | Solvent | 1 | 42.1350 | | 42.1350 | 3.25 | 0.0866 | No |
|  | Temperature | 1 | 226.204 | | 226.204 | 1.74 | 0.2016 | No |
|  | pH | 1 | 240.801 | | 240.801 | 1.86 | 0.1882 | No |
|  | Model | 3 | 888.355 | | 296.118 | 2.28 | 0.1102 | No |
| Psoralen | Solvent | 1 | 235.422 | | 235.422 | 1.334 | 0.0016 | Yes |
|  | Temperature | 1 | 4,053.282 | | 4,053.282 | 229.61 | <0.0001 | Yes |
|  | pH | 1 | 665.667 | | 665.667 | 3.771 | <0.0001 | Yes |
|  | Model | 3 | 4,954.371 | | 1,651.457 | 93.55 | <0.0001 | Yes |
| Bergapten | Solvent | 1 | 28.017 | | 28.017 | 70.93 | <0.0001 | Yes |
|  | Temperature | 1 | 355.267 | | 355.267 | 899.41 | <0.0001 | Yes |
|  | pH | 1 | 117.600 | | 117.600 | 297.72 | <0.0001 | Yes |
|  | Model | 3 | 500.883 | | 166.961 | 422.69 | <0.0001 | Yes |

p > 0.05 = not significant; p ≤ 0.05 = significant; p ≤ 0.001 = highly significant.

**Figure S2.** Phylogenetic reconstruction of AChE orthologs inferred using the Neighbor-Joining (NJ) algorithm based on evolutionary distances derived from the BLOSUM62 matrix.


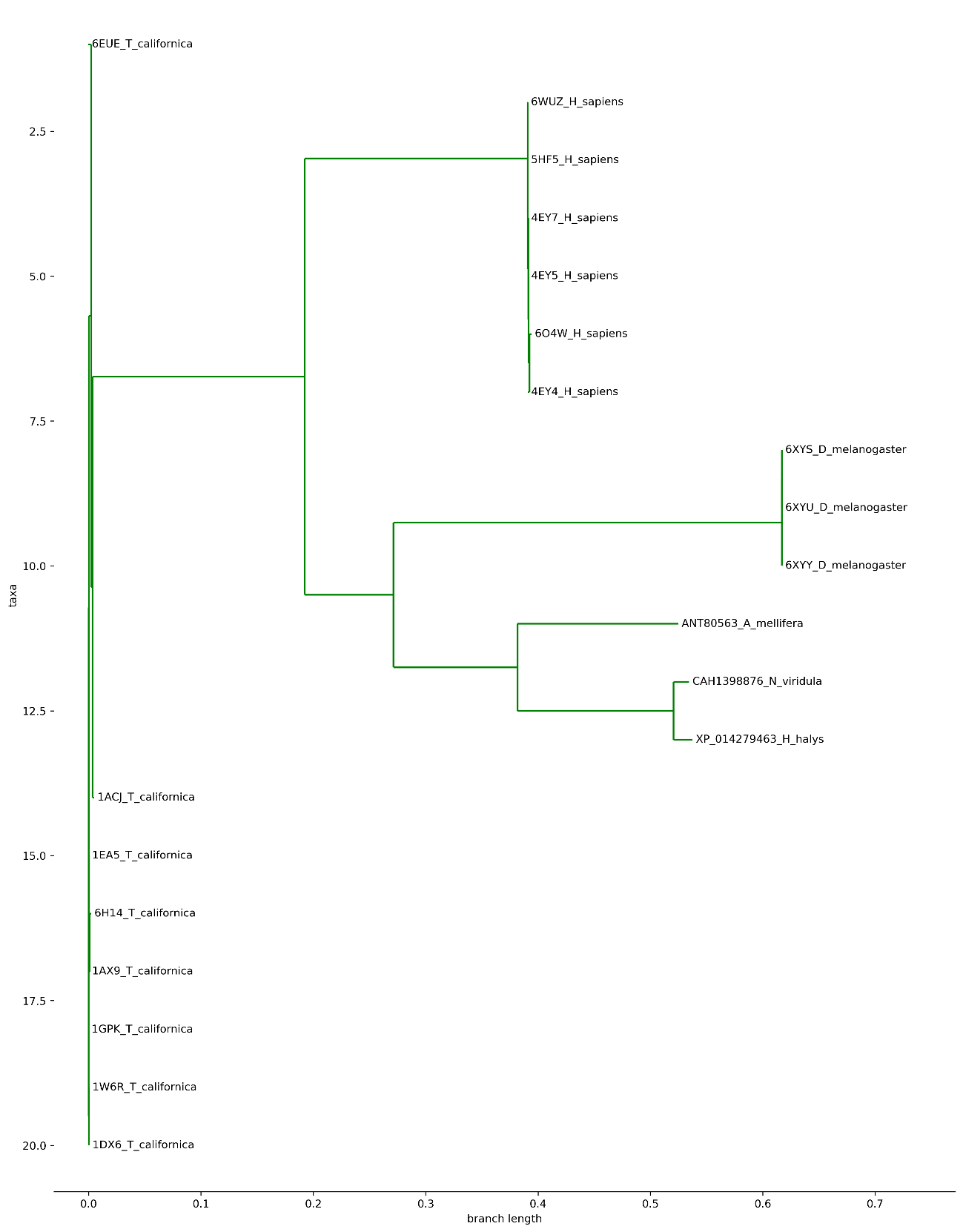


**Table S3**. Pairwise amino acid sequence identity matrix for AChE orthologs.

| **Organism** | ***A. mellifera*** | ***N. viridula*** | ***H. halys*** | ***D. melanogaster*** | ***H. sapiens*** | ***T. californica*** |
| --- | --- | --- | --- | --- | --- | --- |
| *A. mellifera* | 100 | 67 | 67 | 40 | 47 | 47 |
| *N. viridula* | 67 | 100 | 96 | 40 | 47 | 44 |
| *H. halys* | 67 | 96 | 100 | 39 | 46 | 44 |
| *D. melanogaster* | 40 | 40 | 39 | 100 | 38 | 39 |
| *H. sapiens* | 47 | 47 | 46 | 38 | 100 | 58 |
| *T. californica* | 47 | 44 | 44 | 39 | 58 | 100 |

**Table S4.** Pairwise amino acid sequence similarity matrix for AChE orthologs.

| **Organism** | ***A. mellifera*** | ***N. viridula*** | ***H. halys*** | ***D. melanogaster*** | ***H. sapiens*** | ***T. californica*** |
| --- | --- | --- | --- | --- | --- | --- |
| *A. mellifera* | 100 | 89 | 90 | 71 | 76 | 77 |
| *N. viridula* | 89 | 100 | 99 | 72 | 76 | 78 |
| *H. halys* | 90 | 99 | 100 | 72 | 76 | 78 |
| *D. melanogaster* | 71 | 72 | 72 | 100 | 69 | 71 |
| *H. sapiens* | 76 | 76 | 76 | 69 | 100 | 85 |
| *T. californica* | 77 | 79 | 79 | 71 | 85 | 100 |

**
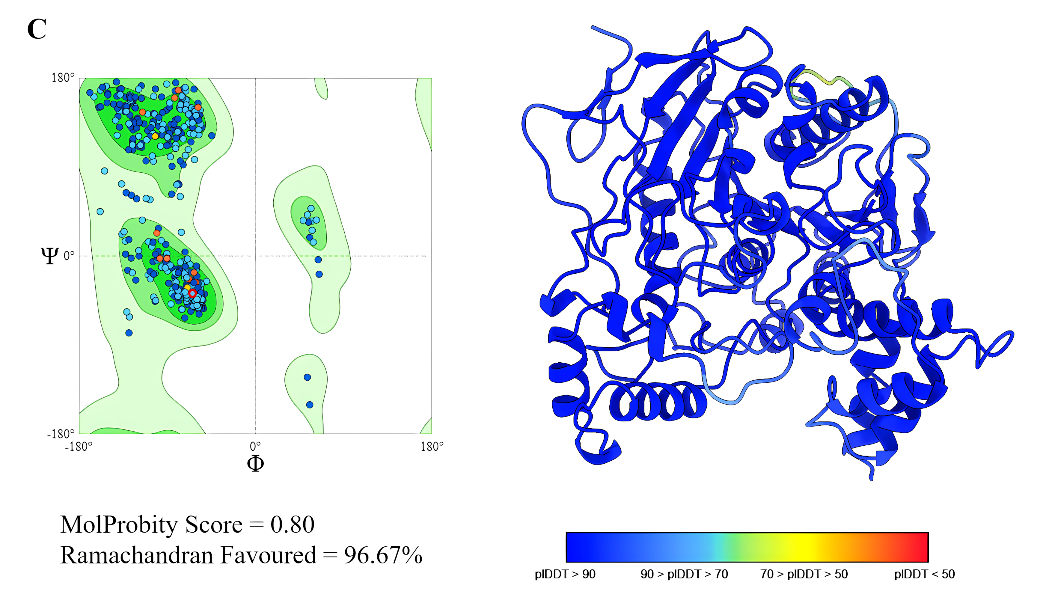

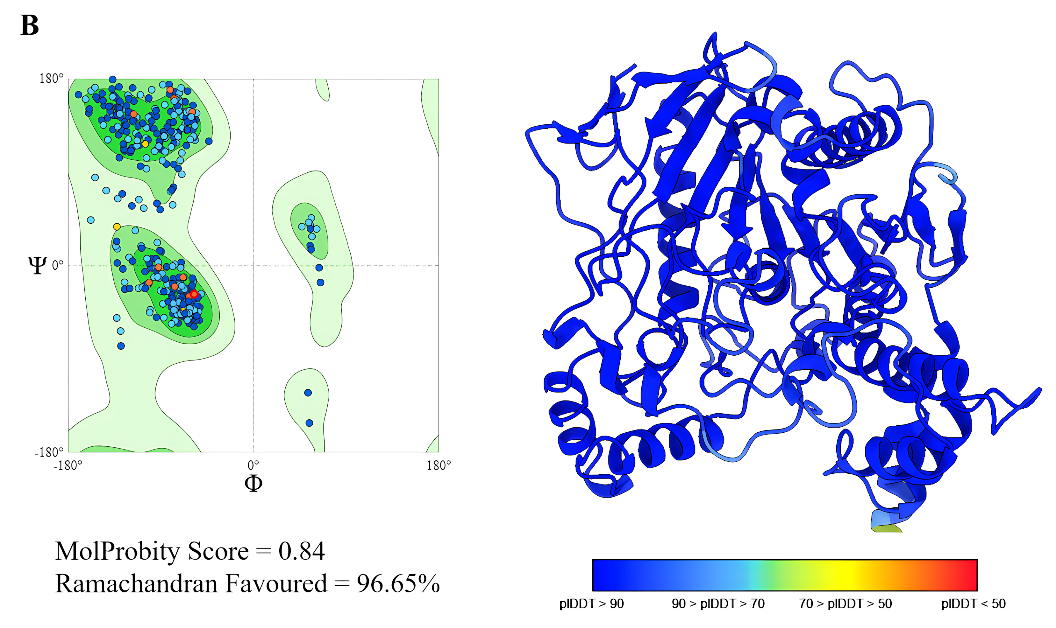

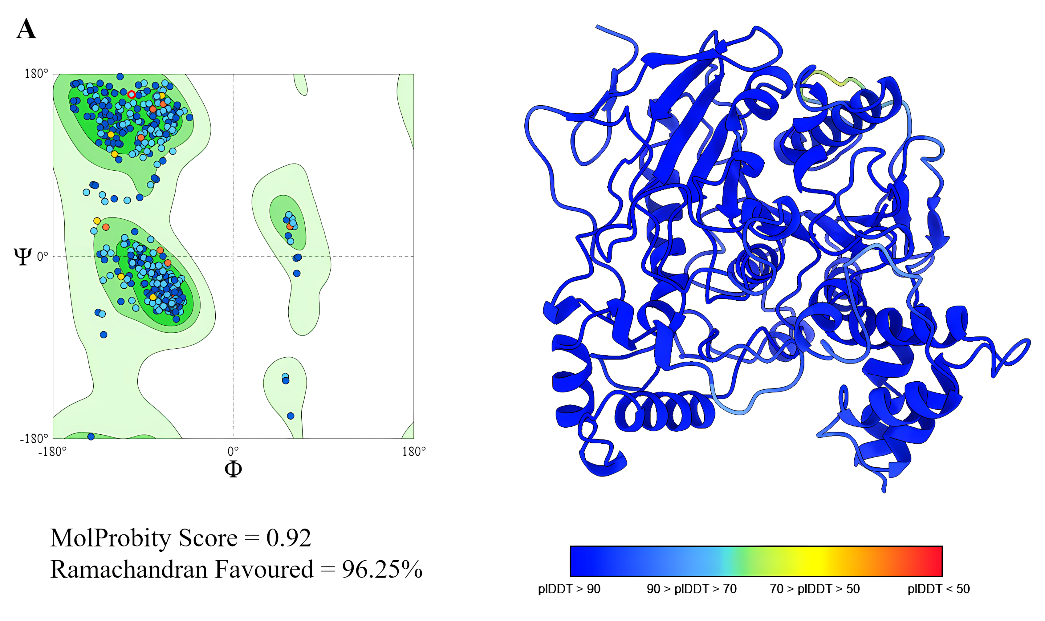
Figure S3.** Structural quality evaluation (Ramachandran plots and pIDDT scores) of the models generated for AChE proteins of *A. mellifera* (A), *N. viridula* (B), and *H. halys* (C).

**Table S5.** RMSD values from the redocking validation protocol comparing predicted binding (blue color) poses against experimental crystallographic conformations (red color).

| **Ligand (PDB ID)** | **Protein (PDB ID)** | **RMSD (Å)** | **Picture** |
| --- | --- | --- | --- |
| E20 | 4EY7 | 0.82 | 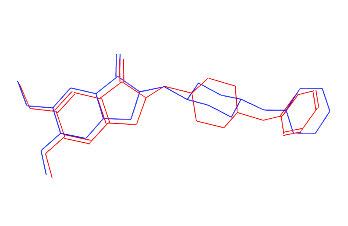 |
|  | 6O4W | 0.82 | 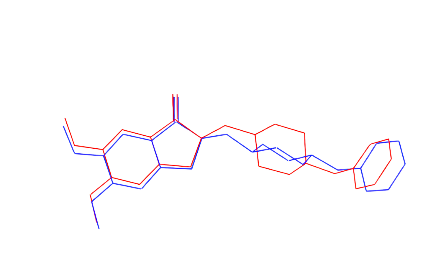 |
| GNT | 1DX6 | 0.54 | 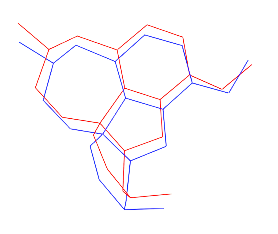 |
| HUP | 1GPK | 2.8 | 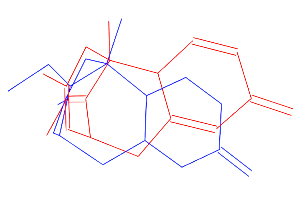 |
|  | 4EY5 | 0.36 | 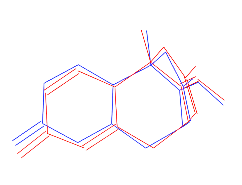 |
| THA | 1ACJ | 0.40 | 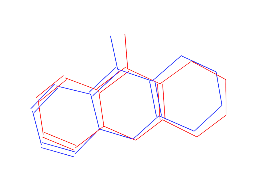 |
| EDR | 1AX9 | 0.33 | 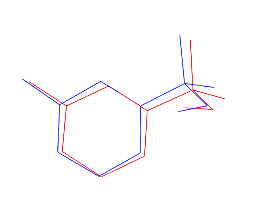 |


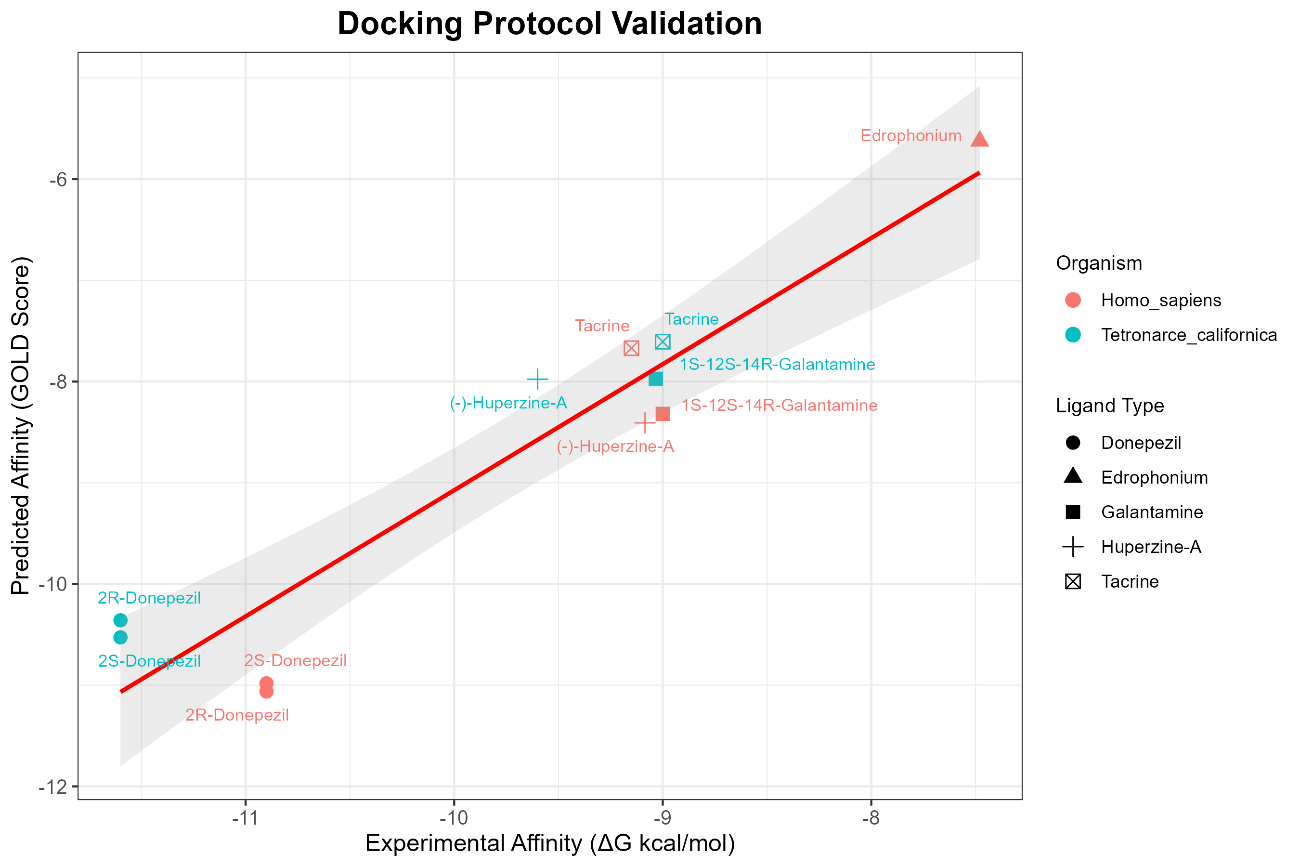
**Figure S4.** Linear regression analysis of the molecular docking protocol validation illustrating the correlation between experimental binding affinities (ΔG) and predicted GOLD fitness scores.
